# Mechanism of Action and Clinical Potential of Fingolimod for the Treatment of Stroke

**DOI:** 10.3389/fneur.2016.00139

**Published:** 2016-08-26

**Authors:** Wentao Li, Haoliang Xu, Fernando D. Testai

**Affiliations:** ^1^Department of Neurology and Rehabilitation, University of Illinois College of Medicine, Chicago, IL, USA; ^2^Department of Pathology, University of Illinois College of Medicine, Chicago, IL, USA

**Keywords:** stroke, neuroprotection, vascular endothelium, fingolimod, FTY720, hemorrhagic stroke, sphingosine-1-phosphate

## Abstract

Fingolimod (FTY720) is an orally bio-available immunomodulatory drug currently approved by the FDA for the treatment of multiple sclerosis. Currently, there is a significant interest in the potential benefits of FTY720 on stroke outcomes. FTY720 and the sphingolipid signaling pathway it modulates has a ubiquitous presence in the central nervous system and both rodent models and pilot clinical trials seem to indicate that the drug may improve overall functional recovery in different stroke subtypes. Although the precise mechanisms behind these beneficial effects are yet unclear, there is evidence that FTY720 has a role in regulating cerebrovascular responses, blood–brain barrier permeability, and cell survival in the event of cerebrovascular insult. In this article, we critically review the data obtained from the latest laboratory findings and clinical trials involving both ischemic and hemorrhagic stroke, and attempt to form a cohesive picture of FTY720’s mechanisms of action in stroke.

## Introduction

Fingolimod (FTY720) is an orally bio-available immunomodulatory drug unique for its reversible leukocyte sequestration properties. In 2010, it was approved by the FDA for the treatment of multiple sclerosis (MS) (1, 2).

Given the current understanding of the role of the immune system in the pathophysiology of brain injury in cerebrovascular diseases, there is now significant interest in the potential benefits of FTY720 on stroke outcomes. Several groups have independently evaluated its effects in rodent models of brain ischemia and intracerebral hemorrhage (ICH). More recently, pilot clinical trials have been conducted demonstrating promising results, albeit in small populations (3–5). Taken together, these studies seem to indicate that administration of FTY720 results in overall improved functional recovery in different stroke subtypes.

The precise mechanisms behind these beneficial effects are still under investigation. FTY720 is a partial sphingosine-1-phosphate (S1P) agonist with immunomodulatory properties that regulates cerebrovascular responses, blood–brain barrier (BBB) permeability, and central nervous system (CNS) cell survival (6, 7). In this article, we will organize these elements and attempt to form a cohesive picture of FTY720’s mechanisms of action in stroke, and critically review the data obtained from recent clinical trials.

## Role of the Immune System in Stroke Progression

Immunomodulation is a well-characterized effect of FTY720 and is thought to mediate some of the beneficial effects seen in stroke models. In order to understand the extent to which the immune system impacts the evolution of stroke outcomes, we will sketch a proposed model of the immune cascade following ischemic insult.

In the immediate aftermath of an ischemic event, complement activation, clot formation, and oxidative stress result in direct damage to local vasculature. Endothelial cells die and detach, interrupting the integrity of the BBB and exposing sub-endothelial antigens. Immune cells adhere to the vessel wall and upregulate the expression of chemoattractant and adhesion molecules that lead to the infiltration of the brain parenchyma by the innate immune system.

A combination of neutrophils, monocytes, and macrophages, this innate immune system further contributes to vascular compromise and early inflammation. One well-documented process is through the release of matrix metalloproteinases (MMPs) by the immune cells, particularly MMP-9, which contributes to the breakdown of the BBB with the resultant edema and growth of the infarcted area (8).

In the parenchyma, glial cells are also activated by the inflammation and damage-associated molecular patterns (DAMPs) released from dying neurons. These reactive astrocytes and microglia further stimulate the recruitment of leukocytes, which release their own pro-inflammatory chemokines, perpetuating a cycle of vascular damage, inflammation, and cell death (9).

The second, adaptive phase of the immune response is mediated predominantly by effector T cells, which are stimulated by DAMPS and brain-specific antigens released upon neuronal cell death (10). These T cells mobilize to the injured regions of the brain, infiltrating a compromised BBB to release pro-inflammatory cytokines, including IFN-γ, resulting in a delayed neurotoxic effect (11, 12). Of note, brain-specific antigens were identified in cervical nodes and palatine tonsils of animals with cerebral ischemia and stroke survivors. Interestingly, some of these antigens were associated with infarct volume and survival. While these studies need to be replicated in larger cohorts, they suggest the participation of peripheral lymphoid tissue in stroke-associated inflammation and outcomes.

Lastly, the inflammatory process is brought to an end via a combination of B cells and regulatory T cells. The latter acts through IL-10, which in combination with TGF-β produced by local macrophages, suppresses further helper T-cell-induced inflammation, and promotes the regeneration of remaining viable neurons (13, 14).

A reduction in various components of the innate and adaptive immune response have been associated with better stroke outcomes. Clinically, a lower ratio of CD14+ pro-inflammatory monocytes to CD16+ reparative monocytes has been correlated with better acute and long-term functional outcomes (15). Similarly, decreased complement activation, specifically the reduced expression of C3, C4, and C-reactive protein, has been associated with better recovery at 3 months post-stroke (16). Experimentally, inhibition of CD8+ and CD4+ T-cell migration into the CNS and direct disruption of CD8+ cytotoxicity has led to reduced infarct volume and post-ischemic inflammation (17). Disruption of the DAMPs-activated γδT cells have also resulted in decreased infarct size and better functional recovery in mice (18). Finally, direct delivery of B cells and IL-10 to the brain in animal models have resulted in a reduction of inflammatory cytokines produced by effector T cells and a reduction of infarct size (19, 20).

## Fingolimod and Stroke-Related Mechanisms of Action

### Pharmacology

**Figure 1:**
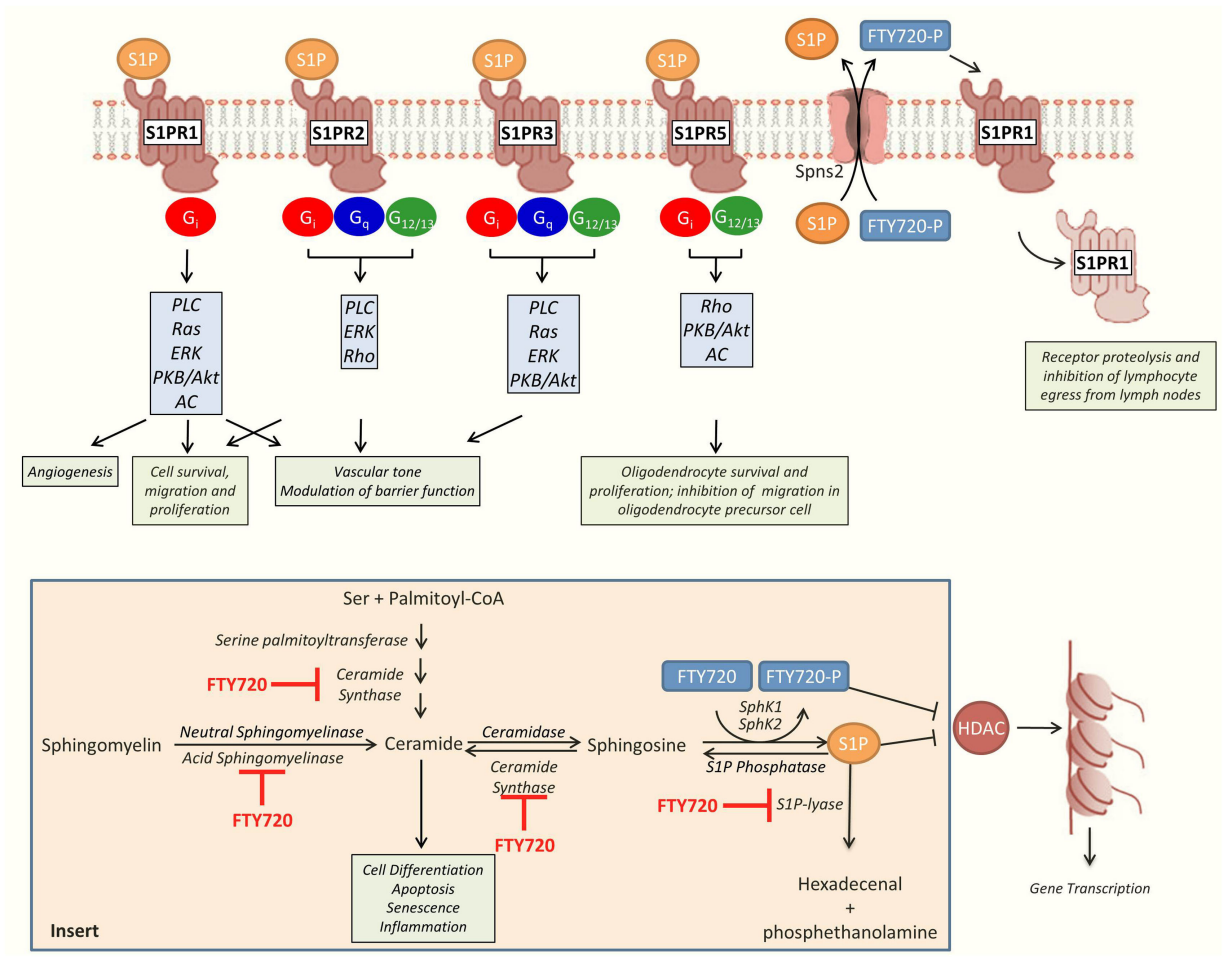
**Overview of sphingosine-1-phosphate (S1P) and fingolimod (FTY720) effects**. S1P signals through five different G protein-coupled cell surface receptors (S1PR 1-5). S1PR1–3 are ubiquitously expressed throughout the body. In comparison, S1PR4 is predominantly expressed in the immune system and S1PR5 in the central nervous system (CNS) and the spleen. These receptors are associated with different profiles of Gα subunits, including G_i_, G_q_, and G_12/13_. The profile of S1PR and Gα subunit expression enable cells to activate different downstream signaling pathways and to orchestrate complex responses in response to single agonist. The activated form of FTY720, FTY720-phosphate (FTY720-P), is a partial agonist of all the S1PRs, with the exception of S1PR2. Downstream, S1PRs regulate oxidative-stress-induced cell survival, endothelial barrier function, leukocyte migration and taxis, and oligodendrocyte survival and proliferation in the CNS (26, 30, 31). In the presence of FTY720-P, S1PR1 is downregulated from the cell surface impeding the egress of lymphocytes from lymph organs and causing lymphopenia (32). S1P and FTY720-P also regulate gene transcription through non-receptor-related epigenetic mechanisms related to the inhibition of histone deacetylases (HDAC). *Insert*: metabolism of sphingolipids (33). Ceramide, a central hub in the metabolism of sphingolipids, has been associated with cell death, quiescence, and differentiation. It can be generated by the breakdown of sphingomyelin by the acid or neutral sphingomyelinases, or synthesized *de novo* from serine and palmitoyl-CoA by serine-palmitoyl transferase and ceramide synthase. Ceramide can be metabolized into sphingosine which is then phosphorylated into S1P by the sphingosine-kinase 1 (SphK1) or 2 (SphK2). S1P is broken down into hexadecenal and phosphoethanolamine by the S1P-lyase. FTY720 is phosphorylated primarily by SphK2. Both S1P and FTY720-P are transported to the extracellular space by the membrane transporter Spns2 (34). In addition to its effect on cell membrane receptors and HDAC, FTY720 also inhibits several key enzymes involved in the metabolism of sphingolipids, including ceramide synthase, acid sphingomyelinase, and S1P-lyase. AC, adenylate cyclase; ERK, extracellular signal-regulated kinase; PKB, protein kinase B; PLC, phospholipase C.

*Isaria sinclairii*, otherwise known as “winter-insect and summer-plant,” is a fungus that has been used in traditional Chinese medicine for over 300 years. Classically prescribed as a panacea for multiple ailments, it produces an atypical amino acid myriocin (ISP-1) that blocks the synthesis of sphingolipids. This chemical compound has since been modified into fingolimod, also known as FTY720 (21). In Western Medicine, FTY720 initially showed promise in preventing ischemic–reperfusion injury following organ transplant, but failed clinical trials due to the development of acute macular edema in some patients (22, 23). In 2010, it became the first oral disease modifying drug approved for treatment of MS (6). In its base form, FTY720 is an orally bio-available, lipophilic molecule that readily crosses the BBB and steadily accumulates in the CNS white and gray matter (24). It bears a structural similarity to sphingosine and is reversibly phosphorylated primarily by Sphingosine-Kinase 2 (SphK2) and, to a lesser extent, by SphK1 (25). In its activated form, FTY720-phosphate is a sphingosine-1-phosphate (S1P) analog that binds to cell membrane G-coupled S1P receptors (S1PR) S1PR1, 3, 4, and 5, but not S1P2 (26). With the exception of S1PR4, these receptors are ubiquitously distributed in the CNS (Table 1). In addition to its action on cell surface receptors, FTY720 regulates the synthesis of different bioactive sphingolipids and, together with S1P, regulates gene expression via epigenetic mechanisms (Figure 1). The half-life of FTY720 averages ~9 days and its pharmacology is not sensibly affected by age, weight, sex, or ethnicity (27).

**Table 1:** **Distribution and function of S1P receptors in the CNS (28, 29)**.

Cell type	Type of receptor	Function
Neuron	S1P1 = S1P3 > S1P2 = S1P5	Neurogenesis, neural progenitor migration, cell survival, and neurotransmission
Oligodendrocyte	S1P5 > S1P1 = S1P2 > S1P3	Oligodendrocyte precursor cell survival, migration, differentiation, and morphology
Astrocyte	S1P3 > S1P1 > S1P2 > S1P5	Proliferation, migration, gap function communication, and growth factor production
Microglia	S1P1 > S1P2 > S1P3 = S1P5	Pro-inflammatory cytokine production

### Immunomodulation

Immunomodulation, particularly peripheral lymphocyte sequestration, is a well-documented mechanism of FTY720. S1P1-receptors are expressed on CCR-7 positive lymphocytes and play a key role in inflammation, stimulating lymphocyte release from peripheral lymphoid tissues in response to endogenous S1P gradients (35, 36). Lymphocyte S1P1-receptor binding by FTY720 results in a functional antagonism via involution and degradation of this cell surface receptor, ultimately inhibiting lymphocyte recirculation (37). In rodent models using 1 mg/kg of FTY720 at 1-, 24-, and 48-h intervals, 60% peripheral lymphopenia can be achieved within 6 h of first administration with peak suppression lasting for 7–9 days (38). By reducing the quantity of infiltrating lymphocytes, FTY720 effectively decreases the direct neurotoxic effects of the adaptive immune system upon the CNS and reduces secondary ischemic damage caused by cytokine-induced inflammation of the surrounding microvasculature (39–41).

**Figure 2:**
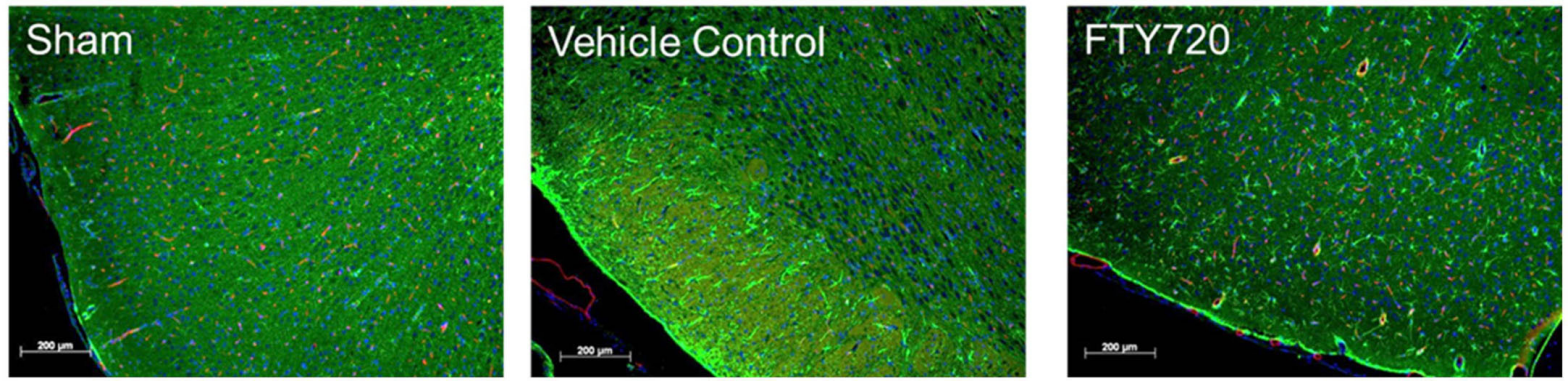
**Effect of fingolimod (FTY720) on cerebral ischemia/reperfusion-associated astrogliosis**. Male adult Sprague-Dawley rats were subjected to middle cerebral artery occlusion (MCAo) for 60 min followed by reperfusion as previously described (45). Three hours after reperfusion, animals were treated with vehicle or 0.5 mg/kg FTY720 intraperitoneally. Brains were harvested at 48 h after surgery, fixed in paraformaldehyde, and mounted to slides. Immunofluorescence staining was performed using anti-glial fibrillary acidic protein antibody (astrocytes; green), anti-endothelial barrier antigen antibody (endothelial cells; red), and DAPI (nuclei; blue) (bar: 200 μm). Representative images obtained for sham, MCAo-vehicle control, and MCAo-FTY720 animals (*n* = 5 per group). A marked increase in astrogliosis was observed in the MCAo-vehicle group, and this was markedly ameliorated in MCAo-FTY720-treated animals.

**Figure 3:**
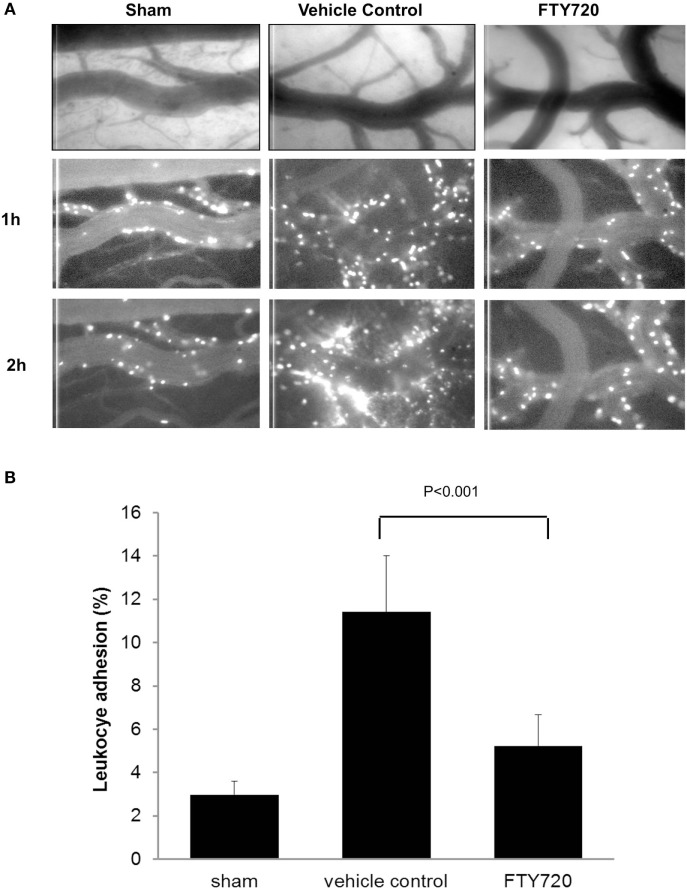
**Assessment of neuroinflammation after cerebral ischemia reperfusion**. Male adult Sprague-Dawley rats were subjected to middle cerebral artery occlusion (MCAo) for 60 min followed by reperfusion. Three hours after reperfusion, animals were treated with vehicle or 0.5 mg/kg FTY720 intraperitoneally. Leukocyte adhesion to pial venules was determined 24 h after reperfusion by intravital microscopy using rhodamine 6G-labeled autologous leukocyte as previously described (46). **(A)** Representative pictures of the vessel anatomy and the trafficking of leukocytes 1 and 2 h after leukocyte labeling with Ro6G in sham, vehicle control, and FTY720-treated animals. **(B)** Quantification of leukocyte adhesion, expressed as the percentage of the vessel area occupied by adherent leukocytes measured 1 h after leukocyte labeling. The MCAo-vehicle group demonstrated a significant increase in vascular leukocyte adhesion at 24 h post reperfusion compared to sham. The treatment with FTY720 decreased the adherence of leukocytes to pial vessels by almost 60%. Significance determined using an unpaired *t*-test. Means ± SD (*n* = 5).

Fingolimod may also attenuate the neuroinflammatory response by acting directly on reactive glial cells, which are activated within minutes following ischemic injury (42). Studies done in rodent models of cerebral ischemia show that FTY720 ameliorates the influx of leukocytes in the ischemic brain and reduces the expression of pro-inflammatory cytokines (IL-1β and IFN-γ) and adhesion molecules, such as intercellular adhesion molecule-1 (ICAM-1) (38, 41). Studies done *in vivo* in our own laboratory confirm that FTY720 reduces leukocyte adhesion to pial vessels and ameliorates astrogliosis in cerebral ischemia–reperfusion models (Figures 2 and 3). In non-stroke models, FTY720 both suppressed TNF-α-induced inflammatory genes and stimulated the production of neurotrophic mediators via S1P1 and S1P3 receptors on astrocytes (43). In particular, FTY720 decreased ceramide production, a pro-inflammatory lipid that causes loss of BBB integrity and increased infiltration of immune cells into the CNS (44). In support of FTY720’s direct CNS effects, direct infusion of FTY720 into rat brains in MS models has been shown to decrease disease severity in the absence of peripheral lymphopenia (7).

### Vasoprotection

It is possible that FTY720 also provides a vasoprotective effect in a manner related to its immunomodulatory action. There is some evidence that FTY720 can induce granulocyte and macrophage colony-stimulating factor (GM-CSF) release from astrocytes, which may limit endothelial cell death after exposure to TNF-α and IFN-γ (47). The presence of astrocyte-derived GM-CSF also suppresses ICAM-1 expression in endothelial cells (48). Decreased surface ICAM-1 reduces leukocyte adhesion to the vessel walls and local platelet activation, which leads to reduced thrombosis and associated inflammation, improving microvascular function (39, 49).

Furthermore, S1P1-receptors are expressed on endothelial cells and administration of FTY720 may directly enhance BBB integrity. First, FTY720 has been shown to directly decrease endothelial ICAM-1 expression in stroke models, which may further contribute to ameliorating the no-reflow phenomenon (41). Second, in non-stroke-related models, FTY720 can stimulate endothelial cells to recruit proteins for adherence junction assembly, reducing vascular permeability and neutrophil infiltration (50). Finally, there is evidence that administration of FTY720 blocks VEGF-induced permeability in human umbilical vein endothelial cells and regulates endothelial cell barrier capacity and vascular permeability in lungs (51). A similar process may also help to maintain the structural integrity of the BBB.

**Figure 4:**
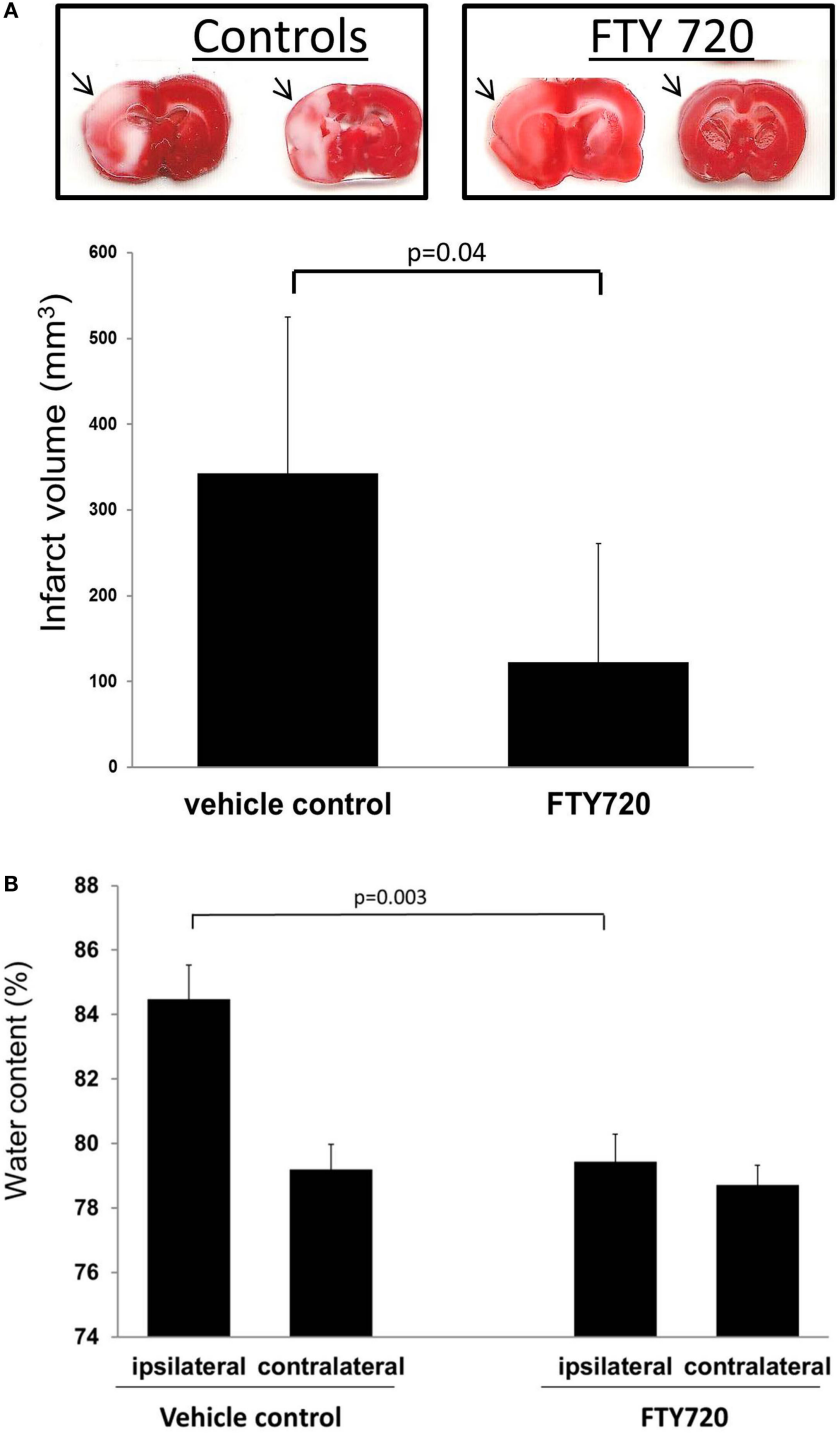
**Infarct volume and brain edema in rats subjected to cerebral ischemia reperfusion**. Male adult Sprague-Dawley rats were subjected to middle cerebral artery occlusion (MCAo) for 60 min followed by reperfusion. Three hours after reperfusion, animals were treated with vehicle or 0.5 mg/kg FTY720 intraperitoneally. Infarct volume was determined as previously described (56). Brain edema was determined by measuring hemispheric water content (41). **(A)** Representative coronal brain cuts obtained 24 h after MCAo. The arrows highlight areas of cerebral infarction. Brain cuts were digitalized and infarct volume determined by using the image analysis software (MetaMorph; Universal Imaging Corp., Downingtown, PA, USA). **(B)** Brain edema determined in the ipsilateral and contralateral hemisphere. Compared to vehicle animals, the treatment with 0.5 mg/kg FTY720 reduced infarct volume and brain edema. Significance determined using an unpaired *t*-test. Means ± SD (*n* = 5).

Taken together, these immunomodulative and vasoprotective mechanisms may contribute to FTY720’s ability to decrease secondary edema formation following cerebral ischemia (41). Such a process would also be consistent with our rat model of middle cerebral artery (MCA) ischemia–reperfusion, where FTY720 treatment resulted in decreased ipsilateral brain edema compared to the vehicle group (Figure 4). Vasoprotection may also account for reduction in hemorrhagic transformation after delayed recombinant tissue plasminogen activator (tPA or alteplase) administration (at 3 h post-stroke), at least in moderate sized thromboembolic strokes (52, 53).

### Direct Neuroprotection

In addition to glial cells, S1P-receptors are ubiquitous within the CNS, with multiple receptor subtypes present on neurons and oligodendrocytes (28). Experimental evidence has shown that fingolimod has direct effects on astrocytes, microglia, oligodendrocytes, neurons, and BBB endothelial cells, which are all affected after stroke (Table 2) (7). There is also increasing evidence that FTY720 may achieve some of its protective effects by directly interacting with neuronal S1P1 and S1P3 receptors, promoting production of anti-apoptotic factors and increasing resilience against ischemic injury. One proposed mechanism of direct neuroprotection is via the S1P/Phosphatidylinositol-3-Kinase (PI3K)/Akt/FOXO3a axis. Administration of FTY720 results in phosphorylation of Akt, which in turn prevents activation of FOXO3a, a transcription factor critical in mediating oxidative stress-induced cell death. In ischemic conditions, this suppression of FOXO3a by FTY720 may contribute to continued neuronal survival (54). FTY720 may also provide a neuroprotective effect through the ERK/bcl-2 pathway. Administration of FTY720 results in persistent ERK phosphorylation in the face of ischemic insult, resulting in enhanced levels of the anti-apoptotic protein Bcl-2, thus rescuing neurons from apoptosis (55).

**Table 2:** **Central nervous system effect of fingolimod (7)**.

Cell type	Function
Neuron	Protects from excitotoxic death
	Restores neuronal function
Oligodendrocyte	Increases survival of oligodendrocyte precursor cells
	Enhances remyelination
	Regulates migration, differentiation, and process dynamics
Astrocyte	Inhibits pro-inflammatory cytokine production
	Inhibits astrogliosis
	Regulates cell migration
Microglia	Reduces microglial activation
	Ameliorates microgliosis
Blood–brain barrier	Reduces blood–brain barrier leakiness

Pretreatment with FTY720 at 48 h before MCA ischemia–reperfusion also leads to an ischemia tolerant phenotype in mice models mimicking the effect of hypoxic preconditioning and decreasing subsequent brain ischemia volume by 33%. This mechanism is hypothesized to act via a hypoxia-inducible factor (HIF), SphK2/S1P1-receptor, and chemokine (C–C motif) ligand-2 (CCL2) signaling pathway, ultimately resulting in increased CCL2 expression in neurons (57). CCL2 is a leukocyte-derived pro-inflammatory chemokine shown to be protective against ischemia in the myocardium (58). It is possible that prophylactic treatment with FTY720 leads to early exposure to low doses of CCL2, which may prime neurons against future ischemic damage, however, the exact mechanism remains to be fully elucidated (59).

Finally, S1P and FTY720 have intracellular effects that are not mediated by cell surface receptors (60). One identified mechanism is the epigenetic regulation of histone acetylation. Both agents are class I histone deacetylase (HDAC) inhibitors and elevated levels of intranuclear S1P or phosphorylated-FTY720 increase acetylated histone H3 (61). This epigenetic phenomenon has been associated with alterations in hippocampal neurons and rescuing memory deficits independent of FTY720’s immunosuppressive actions (62). The beneficial effect of fingolimod on cognition has also been shown in experimental models of neurodegeneration and neuroinflammation, and in memory performance after cerebral ischemia–reperfusion (63–66). However, whether this effect is related to epigenetic mechanisms is yet to be determined.

## Fingolimod in Clinical Trials

Given the consistent benefits demonstrated in laboratory stroke models and the favorable safety profile observed in clinical trials involving MS patients (67), some research groups have started to assess FTY720 in stroke subjects. So far, the results of three small-sized studies have been published by the Tianjin Neurological Institute looking at patients with anterior circulation acute ischemic strokes (AIS) and ICH (3–5).

### Ischemic Stroke

Two small clinical trials investigated the effect of FTY720 in acute cerebral ischemia. The first study published in 2014 was an open-label, evaluator-blinded, non-randomized study that examined the effect of FTY720 on ischemic stroke compared to standard of care alone. The inclusion criteria included age >18 years, National Institute of Health Stroke Score (NIHSS) score at baseline ≥5, anterior circulation ischemic stroke, and time of onset to admission within 4.5–72 h. Major exclusion criteria included history of bradyarrhythmia or atrioventricular blocks, use of antineoplastic or immunomodulating therapies, and macular edema. A total of three consecutive oral doses of FTY720 0.5 mg daily were administered to the study group. The first dose of the study drug was given 1 h after the baseline MRI and no later than 72 h after symptoms onset. The primary endpoint was lesion size at 7 days and microvascular permeability and clinical improvement. Clinical outcomes included (a) NIHSS which is a stroke severity score that ranges from 0 to 42, with higher numbers representing increased stroke severity; (b) modified Barthel index (mBI) which is a validated score of dependency; this score runs from 0 to 100 with higher numbers denoting better outcome with increasing independency to perform activities of daily living; and (c) modified Rankin Scale (mRS) which is a tool that assesses disability; this scale ranges from 0 to 6, with 0 denoting perfect health and 6 death.

A total of 22 patients were assigned to the control or the FTY720 groups. The treatment with fingolimod was initiated at an average of 20 h after stroke onset. Both the control (*n* = 11) and the study groups (*n* = 11) had comparable baseline characteristics. Compared to the lesion volume and location-matched control group, there was no significant decrease in lesion size at 7 days on FLAIR imaging (70 ± 26 vs. 85 ± 30 ml, *P* = 0.69). However, lesion expansion was more commonly seen in the controls (9 ± 3 vs. 27 ± 8 ml, *P* = 0.0494) and infarct volume extension ratio, defined as the ratio of infarct volume change from baseline to day 7/infarct volume at baseline, was significantly decreased in the FTY720 group (15.2 ± 4.2 vs. 41.6 ± 4.5, *P* = 0.0003). In addition, microvascular permeability, measured as parenchymal enhancement contrast-enhanced T1-weighted imaging, was 2× higher in controls compared to the FTY720-treated group (*p* = 0.005). In terms of functional outcomes, study patients demonstrated significant improvement in neurological functions within the first week. This was again observed at 90 days post stroke, with significantly lower NIHSS scores (1.7 ± 0.8 vs. 5.8 ± 1.7, *P* = 0.02) and significantly higher mBI (62 ± 11 vs. 87 ± 8, *P* = 0.0049). In addition, decreased neurological disability, defined as a mRS score of 0–1 at 90 days, was more commonly seen in the FTY720 groups than in controls (0 vs. 73%; *P* = 0.009). In terms of safety, the treatment with FTY720 resulted in a sustained lymphopenia that persisted for more than 7 days and resolved by day 30. Significantly, the rate of complications and adverse reactions, including bradycardia, macular edema, or infections, were similar in the control and the study groups.

In 2015, a second randomized, open-label, evaluator-blinded study was conducted to assess the efficacy of FTY720 in conjunction with tPA. The inclusion criteria included age 18–80 years, first-ever hemispheric stroke due to anterior or middle cerebral artery occlusion (MCAo), and initial NIHSS score ≥5. Patients were excluded from the study if they had contraindications to tPA, pre-existing disability, history of bradyarrhythmia or atrioventricular blocks, concurrent use with antineoplastic or immunomodulating therapies, and macular edema. The first dose of fingolimod was given before alteplase administration. Similar to the previous study, a total of three doses of 0.5 mg oral fingolimod were given in consecutive days. Lesion size, hemorrhagic volume, and NIHSS scores at 24 h were the primary endpoints; also assessed were lesion size at day 7 and clinical improvement up to day 90.

A total of 47 patients were randomly assigned to receive tPA alone or tPA plus FTY720. The mean time from stroke onset to initiation of treatment was ~3 h. The baseline characteristics of the control and the study groups were comparable with the exception of atrial fibrillation that was more commonly seen in the FTY720 group. Recanalization rates were the same in both groups, but compared to the control group, the combined treatment with FTY720 and alteplase resulted in decreased infarct volume expansion (10.1 ± 1.2 vs. 34.3 ± 10.4 ml; *P* = 0.04), decreased hemorrhagic transformation volume (1.2 ± 0.4 vs. 4.4 ± 1.1 ml; *P* = 0.01), lower rate of parenchymal hemorrhage (0 vs. 36%; *P* = 0.002), and greater improvements in NIHSS scores (4 vs. 2; *P* = 0.02), all at 24 h. At day 7, the combination therapy also demonstrated decreased lesion volume compared to alteplase treatment alone (−2.3 ± 2.7 vs. 12.1 ± 3.7 ml; *P* < 0.01) and greater improvement in the NIHSS score from the presenting stroke (2.5 vs. 1; *P* < 0.01). At 3 months, the rate of patients with good neurological outcome, defined as mRS 0–1, was higher in the group of patients that received FTY720 plus tPA (73 vs. 32%; *P* = 0.01). From the safety stand point, malignant cerebral edema requiring craniotomy occurred in only one patient receiving tPA monotherapy but in none of the subjects in the tPA plus alteplase group. Aside from the expected lymphopenia resulting from the use of FTY720, safety outcomes were comparable in both groups.

During ischemia, lymphocytes interact with endothelial cells and platelets to induce microvascular dysfunction and enhanced thrombosis. This process, referred to as *no reflow*, occurs within a few hours after the ictus and results in the obliteration of small capillaries. In addition, the occurrence of ischemia is associated with a profound local inflammatory response. The term *thromboinflammation* refers to the active interplay that occurs between thrombotic and inflammatory mechanisms. Data obtained in experimental models of cerebral ischemia/reperfusion demonstrate that FTY720 ameliorates the number of lymphocytes in the intracranial vascular compartment and reduces ischemia-associated vascular dysfunction and microvascular thrombosis. Thus, it is conceivable that FTY720 contributes to improved outcome in cerebral ischemia by reducing thromboinflammation (39). In addition, enhanced inflammation has been linked to BBB dysfunction and increased risk of hemorrhagic transformation. Studies done in animal models of MCA thromboembolic occlusion show that FTY720 attenuates BBB dysfunction and hemorrhagic transformation with delayed tPA administration at 3 h post-stroke (53).

### Intracerebral Hemorrhage

Intracerebral hemorrhage constitutes a neurological emergency that carries a mortality of ~50%. Hematoma volume and location, age, and the presence of intraventricular extension are established predictors of outcome. Perihematomal edema occurs within the first 72 h post ictus and contributes to both morbidity and mortality (68). The breakdown of the hematoma attracts immune cells that infiltrate the parenchyma and secrete pro-inflammatory cytokines and MMPs. These mediators disrupt the BBB and facilitate the recruitment of additional leukocytes that results in a vicious cycle that contributes to secondary brain injury. Peripheral immune cells, including T-lymphocytes, have been identified as active contributors to perihematomal edema. Studies done in experimental models show that the treatment with FTY720 ameliorates ICH-associated infiltration of lymphocytes and the expression of adhesion molecules and other cytokines, including intercellular adhesion molecule-1 (ICAM- 1), interferon-γ (INF- γ), and interleukin-17 (IL-17) at 72 h after ICH. This reduction in neuroinflammation was associated with reduced cerebral edema, neuronal apoptosis, and brain atrophy and improved neurological outcome (69–71). These findings supported the notion that fingolimod may be protective in ICH.

The Tianjin group investigated the use of FTY720 in non-traumatic ICH using an open-label evaluator-blinded case-control study. The inclusion criteria included supratentorial cerebral hemorrhages between 5 and 30 ml, Glasgow Coma Scales >6, and symptom onset within 72 h prior to admission. Patient were excluded from the study if they had dysphagia, nausea or vomiting, hematoma expansion, pre-existing disability (defined as mRS > 1), bradycardia or atrioventricular block, secondary ICH, macular edema, or concomitant use of antineoplastic or immunomodulatory treatment. Patient were matched by NIHSS as well as hematoma volume and location and then randomly assigned to the FTY720 or control groups. Primary endpoints included perihematomal edema volume at 7 and 14 days (defined by MR imaging), and functional recovery at 90 days. Clinical management was left to the discretion of the treating physician. A total of 23 subjects met the inclusion and exclusion criteria, 11 of whom received 0.5 mg of FTY720 orally daily for three consecutive days. Mean time from stroke onset to intervention was 20 h, with FTY720 administered roughly 1 h after initial CT evaluation.

Both the control and the FTY720 groups had similar baseline characteristics, with the exception of a higher representation of females in the FTY720 arm. The circulating levels of CD4+ T cells, CD8+ T cells, and CD19+ B cells dropped abruptly after the third dose of FTY720. The initial hematoma volume in both groups was comparable (15.4 vs. 16.7 ml, *P* = 0.68). At 7 days post ictus, the perihematomal edema volume in the FTY720-treated group was significantly lower than that in the control group (47 vs. 108 ml, *P* = 0.04). At 14 days, the FTY720 group continued to have a smaller perihematomal edema volume than the control group but this was not statistically significant (55 vs. 124 ml, *P* = 0.07). The loss of significance at 14 days post ICH may represent a floor effect or lack of power. However, it is important to highlight that 100% of FTY720 patients had a striking functional recovery with a GCS of 15 by day 7 compared to 50% of the controls. The rate of patients with good clinical outcome, defined as an mRS of 0 or 1, was 63% in the FTY720 group and 0% in the control group (*P* = 0.001). In addition, 63% of the subjects treated with FTY720 had reduced dependency after ICH, defined by an mBI score of 95–100, as compared to 0% in the control group. In terms of safety outcomes, a single case of transient asymptomatic bradycardia was reported in the group treated with FTY720. Otherwise, both groups had no major differences in the frequency of infections or cardiac arrhythmias.

It is plausible that the reduction in perihematomal edema observed in ICH patients treated with FTY720 relates to its immunomodulatory effects. However, other mechanisms may contribute to this phenomenon. By inducing cytoskeletal rearrangement and increasing the expression of adhesion molecules, including VE-cadherin and β-catenin, S1P has been shown to enhance endothelial barrier permeability and leukocyte entry (50). In addition, the participation of S1P-signaling pathways in endothelial integrity and BBB function has been described in different scenarios. In the rodent model of experimental autoimmune encephalomyelitis, for example, treatment with fingolimod modified the expression of MMPs resulting in a proteolytic balance favoring BBB integrity; and in experimental cerebral malaria, treatment increased serum levels of angiopoietin-1, a marker of endothelium quiescence and stability (72, 73). In addition, FTY720 was shown to block VEGF-induced permeability in human umbilical vein endothelial cells and to regulate endothelial cell barrier capacity and vascular permeability in lungs (51, 52). Whether these mechanisms contributed to the improved outcome observed in ICH patients treated with FTY720 is still to be determined.

## Discussion

Of the small-scale pilot studies described above, larger sample sizes in the setting of double-blinded, fully randomized design would be the next step in validating the results. Better gender balance, racial diversity, as well as homogeneity in lesion location, and neurologic deficit are also worth consideration. Compared to laboratory studies, clinical trials have not shown significant reductions in primary infarct size. Differences in modes of delivery and dosing regimens beyond the 3 days may help assess the ability of FTY720 to achieve reductions in primary lesion size in humans. A larger phase 2 study looking the efficacy and safety of FTY720 in AIS is currently underway. We anticipate that the results of this trial will provide additional valuable data to determine the role of fingolimod in the treatment of this condition (74).

Regarding the concern of suppressing an already compromised immune system during the subacute stage of stroke, stroke induced immunodepression is a well-characterized phenomenon thought to contribute to the high prevalence and morbidity of common infections during ICU and hospital stay (10, 13). So far, however, no significant increase in spontaneous bacterial infections has been reported in either laboratory studies or clinical trials (75).

Finally, in the laboratory studies described above, some contradictory data have not been fully accounted for. Wei et al. were unable to reduce glutamate or hydrogen peroxide-induced cytotoxicity through administration of FTY720 or S1P, which could argue against the direct neuroprotective effects of FTY720 (41). Also, CCL2, which is elevated in neurons by pretreatment with FTY720 and implicated in the development of an ischemia tolerant phenotype in stroke models, is shown to be deleterious when expressed by reactive astrocytes in MS models (76). These and other discrepancies, which will require future verification, may prove to be critical in fully understanding FTY720 and S1P’s mechanism in stroke-related injuries.

## Conclusion

We are just now beginning to understand the role that FTY720 plays in stroke recovery. Taken together, current laboratory and clinical data suggest a beneficial role of FTY720 in preventing secondary progression of tissue injury and promoting short- and long-term rehabilitation, both in AIS with and without tPA, and in ICH. There is also growing evidence supporting the protective effect of fingolimod in subarachnoid hemorrhage (77). Although the exact mechanism is still elusive, that these results are at least in part due to the peripheral sequestration of lymphocytes has been largely accepted. Evidence also exists for other forms of immunomodulation through attenuating reactive astrocytes, vasoprotection (both immune-related and direct), and direct neuroprotection through surface and intracellular S1P receptors.

The sphingolipid signaling pathway modulated by fingolimod have a ubiquitous presence in the CNS, affecting various disease states from Parkinsonism to epilepsy and brain tumors (6, 78). The data presented in this review are predominantly gathered from stroke-related research but includes MS models as well. It is likely that as progress is made in uncovering FTY720 and S1P’s role in other diseases, mechanisms pertaining to stroke will also come to light. In the meantime, we appear to have a drug that continues to show promise in ameliorating the effects of stroke and one that may change the future prognosis and management of cerebrovascular diseases.

## Author Contributions

Dr. WL, HX, and FT participated in the data analysis and interpretation as well as the conception, drafting, and correction of the manuscript. Dr. HX and FT, in addition, participated in data collection. All the authors have revised the manuscript, approved its final version, and confirm the accuracy and integrity of the data included.

## Conflict of Interest Statement

The authors declare that the research was conducted in the absence of any commercial or financial relationships that could be construed as a potential conflict of interest. The reviewer AP and handling editor declared their shared affiliation, and the handling editor states that the process nevertheless met the standards of a fair and objective review.
